# Measuring Spatial Dependence for Infectious Disease Epidemiology

**DOI:** 10.1371/journal.pone.0155249

**Published:** 2016-05-19

**Authors:** Justin Lessler, Henrik Salje, M. Kate Grabowski, Derek A. T. Cummings

**Affiliations:** Department of Epidemiology, Johns Hopkins Bloomberg School of Public Health, Baltimore, Maryland, United States of America; National Institute for Public Health and the Environment, NETHERLANDS

## Abstract

Global spatial clustering is the tendency of points, here cases of infectious disease, to occur closer together than expected by chance. The extent of global clustering can provide a window into the spatial scale of disease transmission, thereby providing insights into the mechanism of spread, and informing optimal surveillance and control. Here the authors present an interpretable measure of spatial clustering, τ, which can be understood as a measure of relative risk. When biological or temporal information can be used to identify sets of potentially linked and likely unlinked cases, this measure can be estimated without knowledge of the underlying population distribution. The greater our ability to distinguish closely related (i.e., separated by few generations of transmission) from more distantly related cases, the more closely τ will track the true scale of transmission. The authors illustrate this approach using examples from the analyses of HIV, dengue and measles, and provide an *R* package implementing the methods described. The statistic presented, and measures of global clustering in general, can be powerful tools for analysis of spatially resolved data on infectious diseases.

## Introduction

The spatial and temporal scales over which cases are likely to be found is one of the most fundamental determinants of the population dynamics of infectious disease, but is also one of the hardest to measure. In particular, heterogeneities in the spatial distribution of the underlying population are often unknown, and may confound the measurement of spatial clustering resulting from the disease process. Here we discuss the potential uses of global clustering statistics in infectious disease epidemiology, and introduce an approach that can be used to measure clustering resulting from the disease process even when information on the underlying spatial distribution of the population is unknown. We discuss the performance characteristics of this approach and present three examples of its use.

## Uses of Spatial Clustering Statistics in Epidemiology

### What is Global Clustering?

Measures of clustering can be broadly divided into two types: local (or first order) clustering statistics that measure the tendency of events (i.e., cases) to occur around a particular point in space, and global (or second order) clustering statistics that measure the tendency of events to cluster in space in general [1]. Both take point pattern data (e.g., the GPS coordinates of case homes) as input. While local clustering statistics have many uses in epidemiology (e.g., developing risk maps [2,3] or identifying individual disease ‘hotspots’ [4]), here we focus on the overall tendency of infectious diseases to cluster in space. These and other terms are further defined in Box 1.

**Box 1. Important terms used in this article.**

**clustering**: points tend to appear closer to each other than would be expected if they were completely spatially randomly distributed.

**regularity**: points tend to appear further away from each other than would be expected if they were completely spatially randomly distributed.

**global clustering statistic**: a statistic that captures the overall tendency of points to occur near other points.

**local clustering statistic**: a statistic that can be used to estimate the expected density of points at specific locations within a study area.

**transmission kernel**: the probability distribution of distances between the location of an infector and infectee.

**positive clustering**: the tendency to see more points of type A near a point of type B.

**negative clustering (i.e., inhibition)**: the tendency to see less points of type A near a point of type B.

**area of elevated risk**: the area around a single case where individuals are more likely to acquire a disease directly from that case (determined by the transmission kernel)

**area of elevated prevalence**: the area around a single case where we expect to see a higher prevalence of cases of the same type

**point pattern data**: data on the spatial location (e.g., GPS coordinates) of persons home, place of likely disease acquisition, or other relevant measure of spatial location.

### The Spatial Scale of Transmission

**Fig 1:**
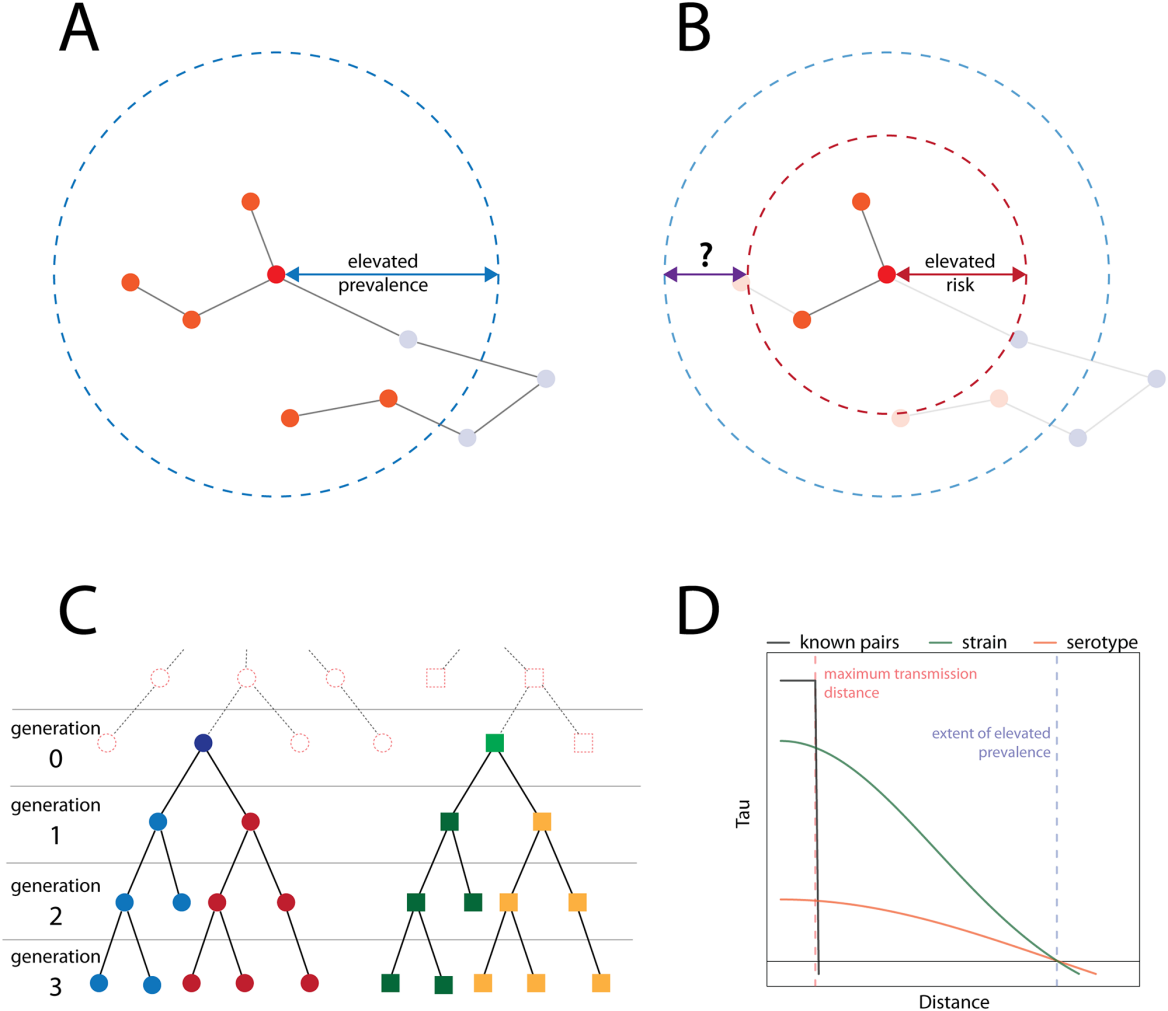
The transmission kernel determines the spatial area where directly transmitted cases may be found (area of elevated risk, red circle in **(B)**). In addition, due to branching in the transmission chain there may be a larger area of elevated prevalence from more distally related cases (area of elevated prevalence, blue circle in **(A)** and **(B)**). **(C)** Example transmission chains. Each color represents a transmission chain of a different strain. The strains are divided into two serotypes, with the blue and red circles representing one serotype and the green and yellow squares a second serotype. **(D)** Increasing ability to discriminate between different transmission chains will increase the power of the τ-statistic to identify the spatial extent of elevated prevalence. Where case-pairs are known, we can identify the area of elevated risk (black line).

The location of a case in relation to their infector defines a spatial transmission kernel for an infectious disease (Fig 1). The shape of this spatial kernel depends on host behavior, mode of transmission and environment. In a directly transmitted disease, such as influenza, the scale of human movement (i.e., host behavior) is the primary determinant of the scale of spatial spread [5]. In contrast, for diseases transmitted through an environmental reservoir (e.g., cholera), by a vector (e.g., malaria) or by intermediary hosts (e.g., West Nile virus), environment and infrastructure may play a more important role [6,7]. The ways in which host, mode of transmission and environment interact to determine the spatial transmission kernel may be complex.

### Observed Clustering of Cases

While a spatial transmission kernel is an essential property of disease transmission, it can be hard to measure. Even if we account for clustering in where people live, the actual distribution of related cases that we see in a population represents the results of multiple generations of transmission. This distribution is driven in part by the transmission kernel for a disease, but can also be highly influenced by both exogenous risk factors and clustering in disease susceptibility [8]. Our ability to limit our analysis to cases that are separated by a larger or smaller number of generations will drive the spatial scale of the clustering observed, as well as how closely this measure mirrors the transmission kernel (Fig 1).

In many cases the observed spatial clustering of cases after multiple generations of transmission may be of greater public health importance than the transmission kernel (Fig 1). If we are attempting to identify cases as part of a response to an ongoing epidemic (i.e., after many generations of transmission), the spatial relationship between linked cases becomes less important that the distribution of cases in general. Similarly, if we aim to intervene against such a disease, the scale of the intervention should match the anticipated distribution of cases [9].

### Clues to Pathogen Epidemiology and Biology

Measures of spatial dependence, particularly when compared across times and settings, may also provide important clues to other aspects of disease epidemiology and biology. Spatial dependence not driven by transmission may indicate clustering in underlying risk factors or behaviors (e.g., clustering of vaccine refusal in the United States [10]). Positive and negative clustering (i.e., inhibition) over time may provide evidence for biological processes where previous infection promotes severe outcomes or there is cross-protection between pathogens [8]. While measures of spatial dependence may lack the inferential power of traditional epidemiologic studies, they can provide important evidence to guide further investigation. The development of global clustering statistics (such as the one proposed here) that exploit information on pathogen genotype or serotype to deal with uncertainty in the underlying population distribution may allow epidemiologic analysis of previously underutilized data sets.

### Some Common Measures of Global Clustering

There exist a number of popular global clustering statistics that either use the exact locations of the points or aggregate them into grid cells. Aggregate (or quadrant) approaches initially place a grid over the study area and count the number of points falling within each grid cell. Area-level statistics can then be used to determine if cells with many points tend to occur near each other (e.g., Moran’s I or Geary’s c) [11,12]. While these methods only have moderate data needs and are simple to implement, they are highly sensitive to the width of the grid cells, do not provide information of the distribution of points within any cell and cannot differentiate between the significant clustering of cells with many points and the significant clustering of cells with few points. An alternative approach is to calculate the mean distance between each point and its closest neighbor. Test statistics exist to assess whether the mean nearest neighbor distance is greater or less than expected in completely spatially random settings [13]. While the nearest-neighbor approaches do not initially require the grouping of points and therefore avoid the loss of information from the aggregation of points, they cannot characterize spatial dependence over different distances. Another distance-based approach, the K-function, by contrast uses estimates of the expected number of points over a range of distances.

$$K\left(d\right)=\lambda^{-1}E\left[N\left(d\right)\right]$$

where *λ* is the spatial intensity of the points in the study area (usually calculated as the size of the study area divided by the number of points) and *E[N(d)]* is the expected number of points within distance, *d*, of a point. The value of the K-function in a homogenous Poisson process is *πd*^*2*^ [14]. Comparisons between an estimated K-function for an observed point pattern and this theoretical value can be used to identify clustering or regularity. Where there is spatial heterogeneity in the underlying population at risk, differences between the K-function of cases and the K-function of a representative sample of the underlying population can be calculated instead. Despite its popularity, K-function values are difficult to interpret using classical epidemiological concepts, and its use is limited to detecting the presence or absence of spatial dependence at varying spatial scales. Because it measures cumulative effects, the K-function can also miss changing patterns in clustering or inhibition [15]. A non-cumulative analogue of the K-function, the pair correlation function, allows finer scale detection of changing patterns in spatial dependence [15]. There also exist methods to estimate the spatial covariance in a point pattern [16, 17]. Inhomogeneous forms of the K-function and the pair correlation function have been developed for situations of large scale differences in where points are located (such as would be generated by different case detection probabilities in different parts of a city), which involve initially estimating the intensity (*λ*) for each location [18]. K-functions and pair correlation functions assume a continuous infinite surface and therefore require edge corrections to account for the often-arbitrary location of study area borders. Versions of the K-function and the pair correlation function also exist for where there are points of different types. This allows us to e.g., estimate the expected number of case of type *j* around each case of type *i* [19]. A more flexible approach, the mark correlation function, incorporates user-defined relationship between types of points, normalizing by the spatial distribution of all points, irrespective of type [20].

## The τ-statistic: An Interpretable Measure of Global Clustering for Infectious Disease

### Goals and Challenges for a Spatial Statistic in Infectious Diseases

If a global clustering statistic is to be useful in the study of infectious disease it must be easily interpretable in terms of disease risk, comparable across settings and distinguish spatial variation due to the transmission process from variation due to clustering in the underlying population. While commonly used techniques like the K-function and Moran’s I are highly useful, none quite satisfy these criteria. In an attempt to meet this challenge, we have sought to develop a measure of spatial dependence that is easily interpretable in terms of disease risk, and extend this measure to make efficient use of pathogen strain (as measured by serotype, genotype, or other measure) to make valid inferences in cases when the spatial distribution of the underlying population is unknown. Hence, this approach should be useful even when the catchment area for cases or the underlying distribution of the population is unknown.

### A Natural Measure of the Clustering of Disease Risk

Epidemiologists typically characterize populations or exposures leading to elevated risk of a health outcome using measure or approximation of relative risk. Measures such as the hazard ratio, incidence rate ratio and the cumulative incidence ratio will be familiar to all epidemiologists. We propose the τ-statistic, a measure of the relative risk of someone at a particular spatial distance from a case also being a case, versus the risk of anyone in the population being a case. That is:

$$\tau \left(d_{1},d_{2}\right)=\frac{\lambda \left(d_{1},d_{2}\right)}{\lambda }$$

where *λ*(*d*_1_, *d*_2_) is the expected incidence rate of someone in distance range *d*_1_, *d*_2_ of a case, and *λ* is the average incidence across the entire population. If the underlying population is known, and the incidence rate is constant over some period, then this can be trivially estimated by:

$$\hat{\tau }\left(d_{1},d_{2}\right)=\frac{\hat{\pi }\left(d_{1},d_{2}\right)}{\hat{\pi }\left(0,\;\infty \right)}$$

where $\hat{\pi }\left(d_{1},d_{2}\right)$ is the estimate probability that a case occurs within some distance range of another case (see S1 Text). This statistic has an easy interpretation, for instance if HIV cases are twice as common within a kilometer of another HIV case as they are in the population in general, then *τ*(0,1km) = 2. Edge corrections are not necessary when calculating this statistic, and the estimator can be easily modified to take into account the likely timing between related cases (see S1 Text).

### Measuring the τ-statistic when the underlying population is unknown

In infectious disease epidemiology, we are often interested in the relative risk of infection at various spatial scales in situations where we do not know the underlying distribution of the population at risk. In these circumstances we can adapt the τ-statistic to use information on the infecting pathogen (such as serotype/genotype) to distinguish between pairs of cases that are consistent with coming from the same transmission chain and pairs that are inconsistent with coming from the same chain. For example, there are four serotypes of the dengue virus that often co-circulate. Pairs of cases infected by the same serotype (homotypic cases) may come from the same chain whereas cases from different serotypes (heterotypic cases) must come from separate chains. Simply approximating *π*(*d*_1_, *d*_2_) by proportion of the cases within a spatial region that are homotypic biases spatial estimates towards the null (see S1 Text), but a valid estimator of the τ-statistic can be achieved by:

$$\hat{\tau }\left(d_{1},d_{2}\right)=\frac{\hat{\theta }\left(d_{1},d_{2}\right)}{\hat{\theta }\left(0,\infty \right)}$$ (1)

where $\hat{\theta }\left(d_{1},d_{2}\right)$ is an estimator of the *odds* that a case within the distance range *d*_1_, *d*_2_ of a case is homotypic (see S1 Text proof). This estimator assumes that the probability of a heterotypic case occurring within *d*_1_, *d*_2_ of a case is the same as the probability of a heterotypic case occurring anywhere in the population. If this is not the case, then Eq (1) is still a valid estimator, but its interpretation changes slightly. It is now a measure of the clustering in potentially related dengue cases *over and above* the clustering of unrelated dengue cases due to secular factors (e.g., environmental conditions). So in this case, a value of τ(0,150m) = 2 for dengue would mean that dengue cases within 150m of one another are twice as likely to be of the same serotype (i.e., potentially related) than any two cases overall. The τ-statistic is robust to heterogeneities in sampling probability over a study area, as the probability of sampling will similarly affect both the numerator and the denominator of the statistic. Similarly, differences in sampling of a particular strain or subtype will not affect the estimate. The maximum likelihood estimator of *τ*(*d*_1_, *d*_2_) is provided (appendices 5, 6 and 7).

### Use of temporal information

Where we do not have information on genotype or serotype, we may be able to use temporal information to distinguish those cases that cannot be closely related from those that may be closely related. A case occurring within a small number of serial intervals (e.g. one to two) from another case has the potential to be that case’s direct offspring or share a recent common ancestor, whereas those cases separated by longer intervals are less likely to be so. In such circumstances, $\hat{\theta }\left(d_{1},d_{2}\right)$ is an estimator of the odds that a case within the distance range *d*_1_, *d*_2_ occurred within a short time frame.

The τ-statistic is equivalent to ratios of particular multitype pair correlation functions. For example, where both a sample of cases (points of type *i)* and a sample of the underlying population are known (points of type *j*), the τ-statistic is a ratio of *g*_*ii*_*(d)* and *g*_*ij*_*(d)* (where *g(d)* is the pair correlation function). The specific forms of the pair correlation functions in the ratio will depend on the type of points in the dataset and the relationship between them (e.g., homotypic).

### Calculation of statistical significance

Simulation methods can be used to calculate statistical significance and confidence intervals for the τ-statistic. The null distribution of the τ-statistic can be determined by repeatedly randomly permuting the locations of the point pattern data (i.e., randomly assigning each covariate vector to one of the existing point locations, one covariate vector per location) and recalculating the τ-statistic after each permutation, then taking quantiles of the resulting distribution as one would do in point pattern data (a similar process has been used to determine the null distribution for other spatial statistics [14]). Similarly, confidence intervals can be calculated using adapted bootstrapping techniques (modifications are needed to avoid comparing bootstrapped cases with themselves when calculating $\hat{\tau }\left(d_{1},\;d_{2}\right)$ see S1 Text).

### R package

We have developed and submitted to CRAN an R package, *IDSpatialStats*, which implements the methods discussed in this manuscript (also available from https://github.com/HopkinsIDD/IDSpatialStats) and includes methods to calculate the τ-statistic for point pattern data and examples of its use.

### Performance of the *τ*-statistic in different settings

Observed cases of disease usually come from multiple generations of transmission stemming from one or more transmission chains that share a common ancestor sometime in the (often very distant) past (Fig 1C). The true spatial transmission kernel is the distribution of distances between directly linked cases. Pathogen typing can give us some ability to distinguish cases that that might come from the same chain of transmission from those that definitely do not. Since spatial dependence should only exist between related cases, and not unrelated or very distantly related cases, comparison of cases that could be possible related with those that are not gives some measure of spatial dependence. As our ability to distinguish cases separated by a small number of generations of transmission from those more distantly related increases, our ability to identify the existence of spatial clustering in transmission increases (Fig 1D). If we had a typing method of sufficient resolution to directly link pairs of cases, the τ-statistic would precisely identify this spatial kernel; but in general we will only have coarse data and will be more closely approximating the area of elevated prevalence (Fig 1).

**Fig 2:**
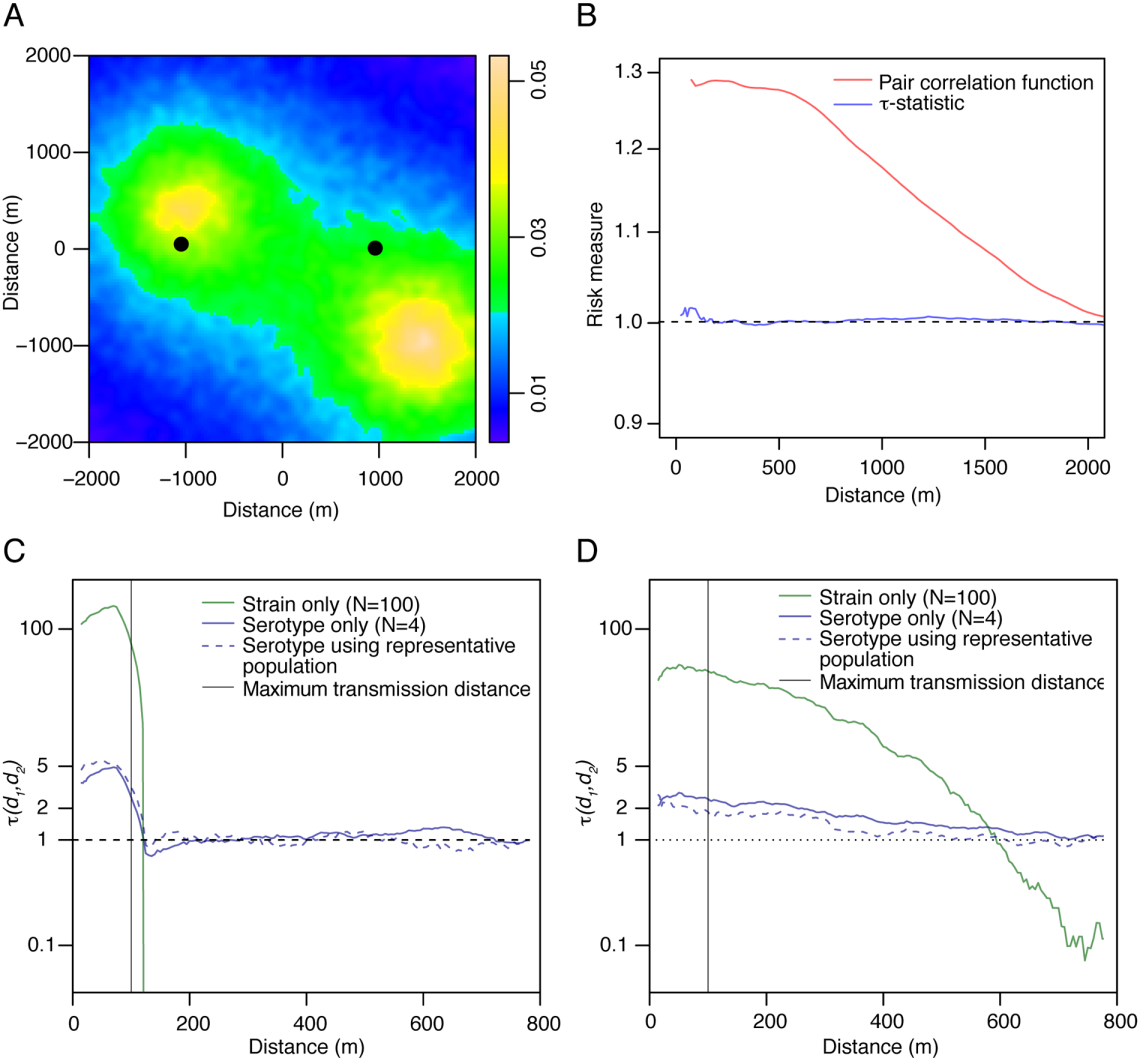
Overview of simulated epidemics of 100 separate transmission chains. Each transmission chain consisted of cases caused by a single strain, with all strains divided into four serotypes. **(A)** Distribution of underlying population for simulated data. The black square represents the area of analysis to avoid edge effects from the simulation process. The two black dots represent two ‘surveillance hospitals’ (relevant for the spatially-biased observation scenario set out in Fig 3). **(B)** Using disease type information (blue) allow the τ-statistic to correctly show no spatial clustering of disease in a spatially clustered population, whereas the pair correlation function would falsely indicate clustering in disease cases. **(C)** τ-statistic results from simulated epidemics where the cases infected individuals between 0 and 100m away. Each case infected one individual (effective reproductive number of one). **(D)** As (C) but each case infects two individuals (effective reproductive number of two).

Using simulated epidemics, we can assess the performance of the τ-statistic in a population that is itself spatially clustered (population distribution shown in Fig 2A). If a disease is distributed homogeneously throughout the population, an estimate of clustering compared to a homogenous Poisson process (e.g., a pair correlation function assuming homogeneous population) would show spatial clustering in the disease on the same scale as the clustering of the population itself (Fig 2B). However, if we can identify potentially related cases through serotype or genotype, the τ-statistic correctly estimates no clustering in the disease process (i.e., that cases near a case of a particular type are no more likely to be of that type than any other case in the population). If we suppose that there is spatial clustering of the disease such that infectious individuals only infect those living within 100m of their home, then the same approach correctly identifies the presence of spatial clustering (Fig 2C and 2D). If the effective reproductive number is approximately 1 (i.e., each infectious case infects approximately 1 other person), as might be true for an endemic disease, then using strain information and restricting comparisons to cases occurring near enough in time to possibly be related, the area where the τ-statistic significantly above 1 precisely estimates the 100m transmission kernel (Fig 1C). If the reproductive number is greater than 1, then the τ-statistic again correctly identifies the presence of clustering, but the distance at which significant clustering occurs is greater than that of the transmission kernel (Fig 2D). This is because multiple cases caused by the same “parent” infector (or grand-parent infector, etc.) will themselves tend to be closer to each other compared to unrelated cases, but the distribution of distances between them will be greater than the actual transmission kernel. This is not to say that the τ-statistic is wrong, rather it estimates the distance of elevated prevalence (which may be of primary interest), rather than the distance of elevated risk (Fig 1). In both cases, as our ability to distinguish strains increases (i.e., as we can identify more distinct lineages), the magnitude of the estimated spatial clustering increases. Importantly, even in scenarios where our ability to identify closely related cases is reduced we still estimate the same scale of transmission, but the magnitude of this estimate decreases (as does the power to detect this difference). Further, in both simulations where we used an R0 of one (Fig 2C) and where we used an R0 of two (Fig 2D), we obtained consistent estimates of spatial dependence when the underlying population was known and the τ-statistic was calculated using a ratio of π’s (dashed blue lines) as when it was not known and the τ-statistic was calculated using a ratio of θ’s (solid blue lines).

**Fig 3:**
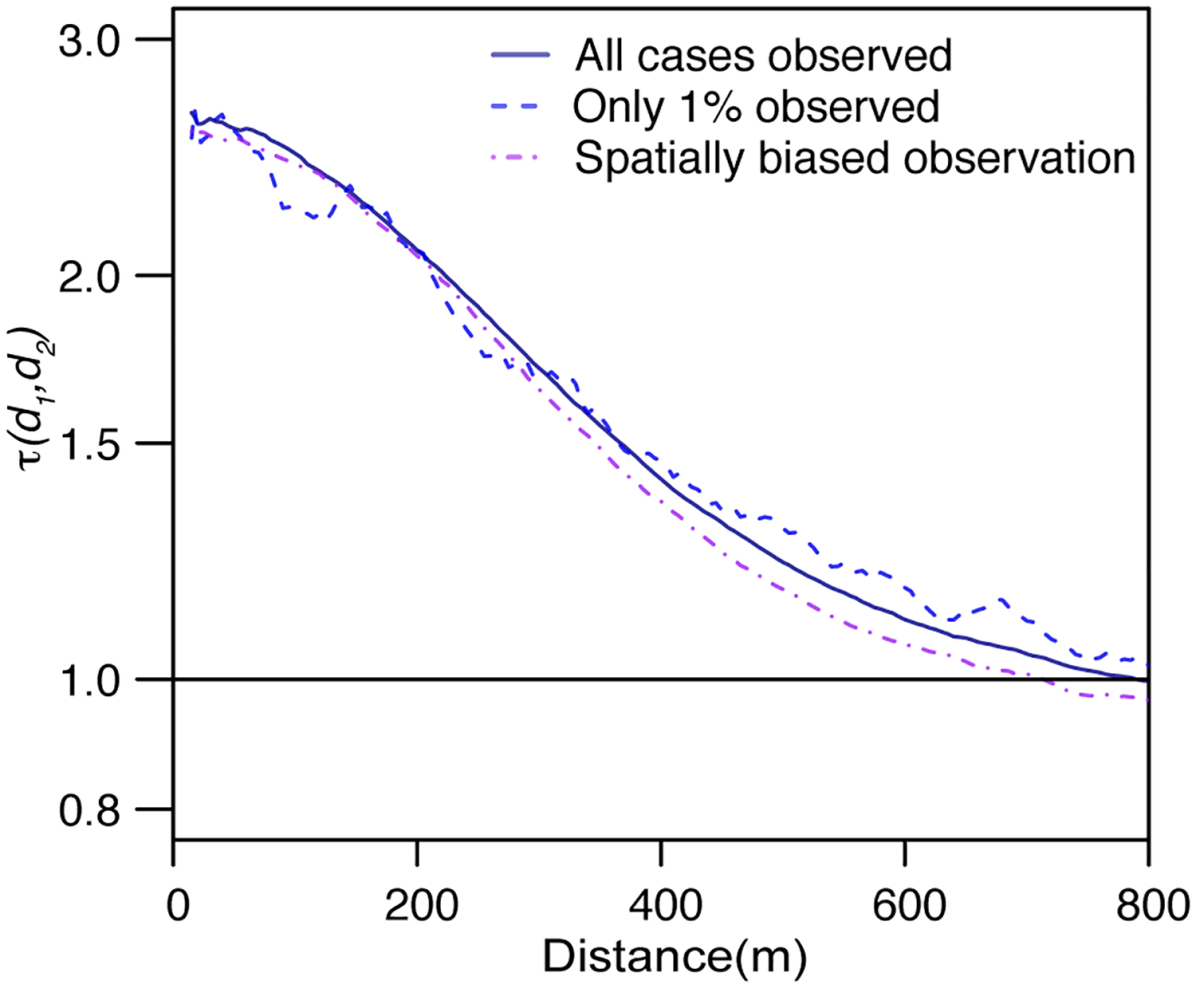
Performance of the τ-statistic under different observation scenarios: complete observation (blue solid line), spatially random observation (dashed blue line) and spatially biased observation (purple line). For the spatially random observation all cases had a 1% probability of being observed. For the partially observed observation, the probability of observation was 0.1xexp(-*d*), where *d* was the distance (in km) to the closest ‘surveillance hospital’ in the area (marked by two black dots in Fig 2A).

We are rarely able to detect all infections in an outbreak. Large numbers of asymptomatic infections and imperfect surveillance systems mean that only a minority of cases may be captured. The τ-statistic is robust even when 99% of cases are unobserved (Fig 3). Further, even when there exists underlying spatial bias in the detection of cases (such as would be expected if only cases from two healthcare providers were analyzed), the τ-statistic provides consistent estimates to when all cases are observed (see S1 Text and S2 Fig for simulation methods and for the results of additional simulations where the healthcare providers are located in other parts of the study area).

## Estimates of the τ-statistic from case data

### Spatial Patterns of Dengue in Bangkok, Thailand

Dengue is a mosquito borne virus that exists in one of four genetically distinct forms called serotypes. The virus causes substantial morbidity and mortality in global tropical and sub-tropical regions. Once a person is infected with a serotype they are immune to that serotype for life, but may still be infected with another serotype [21]. All four serotypes are endemic to Bangkok. The spatial dependence between individuals infected with the virus remains poorly characterized. If we can understand the tendency for individuals infected by the virus to be found near each other, this will aid intervention efforts as well as help elucidate mechanisms that determine pathogen dispersal (e.g., the relative role of human and mosquito movement).

**Fig 4:**
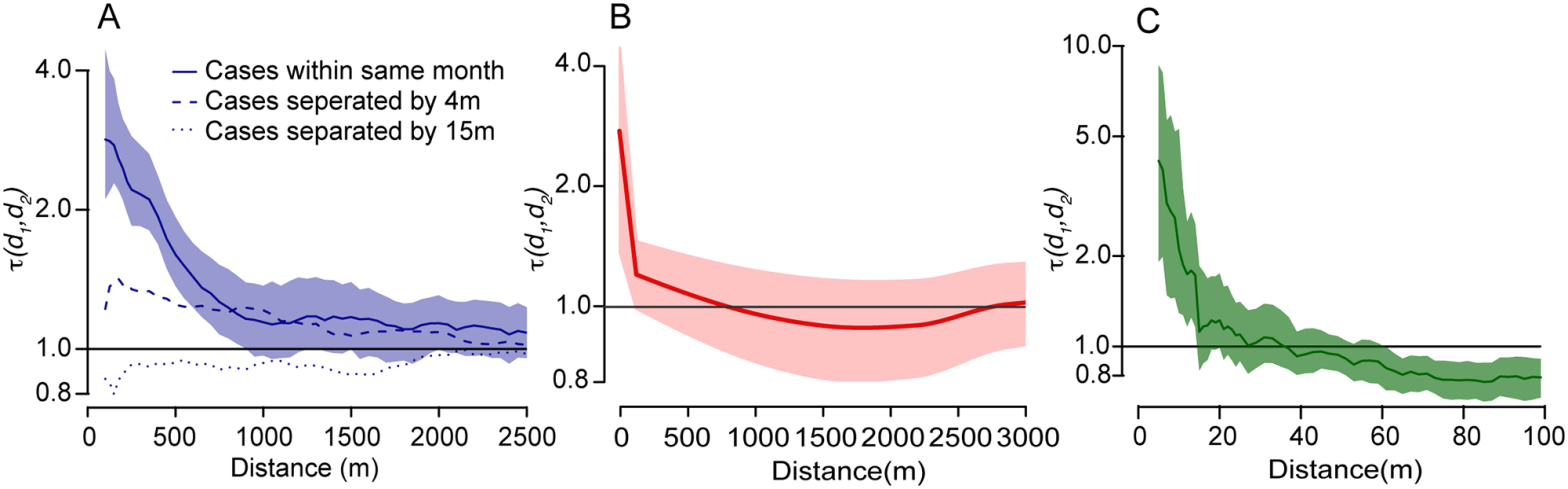
**(A)** τ-statistic results from 1,912 dengue cases that presented at a Bangkok hospital between 1995 and 1999. The solid line represents the probability of cases occurring within the same month being of the same serotype, relative to any two cases being of the same serotype and occurring within the same month. The dashed line is the same analysis but with a temporal lag of four months and the dotted line are the results using a temporal lag of 15 months. Confidence intervals for the temporal lags of four months and 15 months are presented in S1 Fig. **(B)** Smoothed τ-statistic of HIV incident to HIV prevalent individuals in Rakai Uganda. The τ-statistic is calculated within households (i.e, at 0m) and in a sliding 250m window up to the average distance between community households, 3.3km. **(C)** τ-statistic results from 188 measles cases from Hagelloch, Germany in 1861–1862. The τ-statistic is calculated using a sliding 50m window and compares the risk of observing cases at that distance range for individuals sick within two weeks of each other to all time.

To characterize the spatial dependence between cases of dengue in Bangkok, a study used the geocoded home addresses of 1,912 patients that presented at a local hospital with confirmed dengue between 1995 and 1999, the date they were admitted and information on the infecting serotype [8]. The study used the τ-statistic to compare the probability that a pair of cases infected within a defined space-time window were of the same serotype (and therefore consistent with transmission) with the probability that any pair of cases infected within the same time period were infected by the same serotype. In such a way, it was able to capture the tendency that cases that occurred near each other were infected by the same serotype, over and above that expected given the underlying serotype distribution of cases at that time. The study found that cases presenting at the hospital within a month of each other tended to be infected by the same serotype at distances up to around 2 km (Fig 4A).

### The Spatial Scale of HIV Transmission Networks in Rakai Uganda

HIV is endemic in sub-African Africa and occurs with higher incidence than anywhere else in the world; however, the mechanisms of HIV spread and persistence within African populations are poorly understood, particularly at local spatial scales. For instance, the relative frequency of direct transmission between sexual partners who live far apart, versus direct transmission between partners who reside in the same community is unknown. Insights into the spatial scale of HIV transmission can be gained from global spatial clustering analyses. Such spatial analyses can inform basic HIV epidemiology, control efforts, and the design of community randomized clinical trials for HIV prevention, the latter of which often assume that the majority direct transmission occurs within communities.

In a large population-based cohort in rural Rakai, Uganda, HIV incidence was measured in over 8000 geolocated households in 46 communities [22]. The τ-statistic was used to estimate the probability that an individual living within a particular distance of an HIV incident case was also HIV infected relative to the probability that any individual was HIV-infected in the entire study population (Fig 4B). The authors found evidence of significant spatial dependence between HIV incident and HIV infected individuals, prevalent and incident, at spatial scales consistent with household transmission—i.e transmission events in initially HIV discordant or incident HIV concordant cohabitating couples; however, there was no evidence of significant spatial dependence between incident and other HIV-infected persons at spatial scales consistent with community transmission. These findings suggested that, the HIV epidemic in Rakai is characterized by frequent cross-community transmission.

### Measles spread in Hagelloch Germany (1861–1862)

To assess evidence of spatial dependence using the τ-statistic in a directly transmitted disease, we used data from a well-studied outbreak of measles in Hagelloch, Germany in 1861–1962. 188 children became sick with measles between October 1861 and January 1862. Information on the location of the case homes and when they presented with symptoms is available in the R package *surveillance* [23]. In this circumstance, as we do not have information on the infecting pathogen that could allow us to discriminate between pairs that are not transmission related and pairs that could be, we instead used the time difference between case-pairs. The τ-statistic was used to estimate the risk that case-pairs were sick within two weeks of each other (the serial interval for measles), relative to all time periods for different distance ranges, relative to those cases occurring at any time and location during the entire outbreak. We found that individuals living <10m apart were 4.1 times more likely to become sick within two weeks of each other (95% confidence interval: 1.9–8.6) than any two cases overall (Fig 4C). Spatial clustering was observed at distances up to 15m. This is consistent with household/immediate neighbor transmission being an important contributor to the spread of the outbreak, however, not larger distances. These findings are consistent with other studies from the outbreak that found that household transmission and transmission in schools dominated the epidemic (the latter of which would promote transmission over a wider area, not necessarily dependent on where people live)[24].

## Discussion

Global clustering statistics are an important tool for spatial analytics that can be used to better understand the transmission of infectious disease. The τ-statistic presented here represents one approach to measuring global clustering that has an easy interpretation and overcomes many of the challenges encountered when analyzing infectious disease data. To catalyze use of this approach in empirical studies and methodological research, we have provided an open source R package implementing many of the methods presented here (to which all are welcome to contribute). Important areas for further work include, extension of these methods to better analyze particular types of space time data, the development of statistics to derive transmission kernels from observed clustering data, and new techniques for identifying specific clusters building on the ideas presented here.

The τ-statistic provides a valuable tool to capture spatial dependence in epidemiological relevant terms. However, it should be used alongside existing measures of spatial dependence, in particular as it provides a qualitatively different tool to other approaches. This means that they may not be comparable on a quantitative level. Further, we describe the application of the τ-statistic to specifically infectious disease point pattern processes. In these processes, the clustering process for the underlying population is typically independent from the clustering due to disease transmission. Where the underlying population is not known, we expect independence in the clustering processes of the different pathogen types (e.g., independent clustering of homotypic and heterotypic cases of dengue). Such an approach may not be appropriate where we do not have any *a priori* insight into the spatial dependence properties of the different types of points. In such circumstances classical approaches such as differences in K-functions for marked point pattern processes may be preferable. Further, the τ-statistic cannot be used in simple point patterns that do not have additional information attached to points (such as time of infection or pathogen strain). In these circumstances, spatial dependence calculated by K-functions compared to completely spatially random populations could be calculated.

This statistic has already proven useful in the analysis of the spatial dispersal of wide range of infectious diseases, including dengue, chikungunya, influenza and HIV [8,22,25–27]. Though this approach was developed with an eye towards infectious disease, it may also find application in the analysis of many types of epidemiologic data.

## Supporting Information

S1 Fig Confidence intervals for Bangkok dengue example. Confidence intervals for *τ*(*d*_1_, *d*_2_) estimates for cases separated by (A) four months and (B) 15 months. Results from 1,912 dengue cases that presented at a Bangkok hospital between 1995 and 1999.
(TIF)

S2 Fig Sensitivity analysis with different healthcare provider locations. Estimates of *τ*(*d*_1_, *d*_2_) when there is spatially biased observation dependent on the location of a pair of healthcare locations. (A) Locations of pairs of healthcare providers. (B) Estimates of *τ*(*d*_1_, *d*_2_) using locations in (A). The color of each line is the color of the corresponding locations in (A). The solid blue line is the estimate when all cases are observed.
(TIF)

S1 Text Supplementary technical appendices. (PDF)
