# Supplementary figures and images for: Measuring Spatial Dependence for Infectious Disease Epidemiology
Source: PLoS One. 2016 May 19;11(5):e0155249. doi: 10.1371/journal.pone.0155249 (PMC4873007; doi:10.1371/journal.pone.0155249)

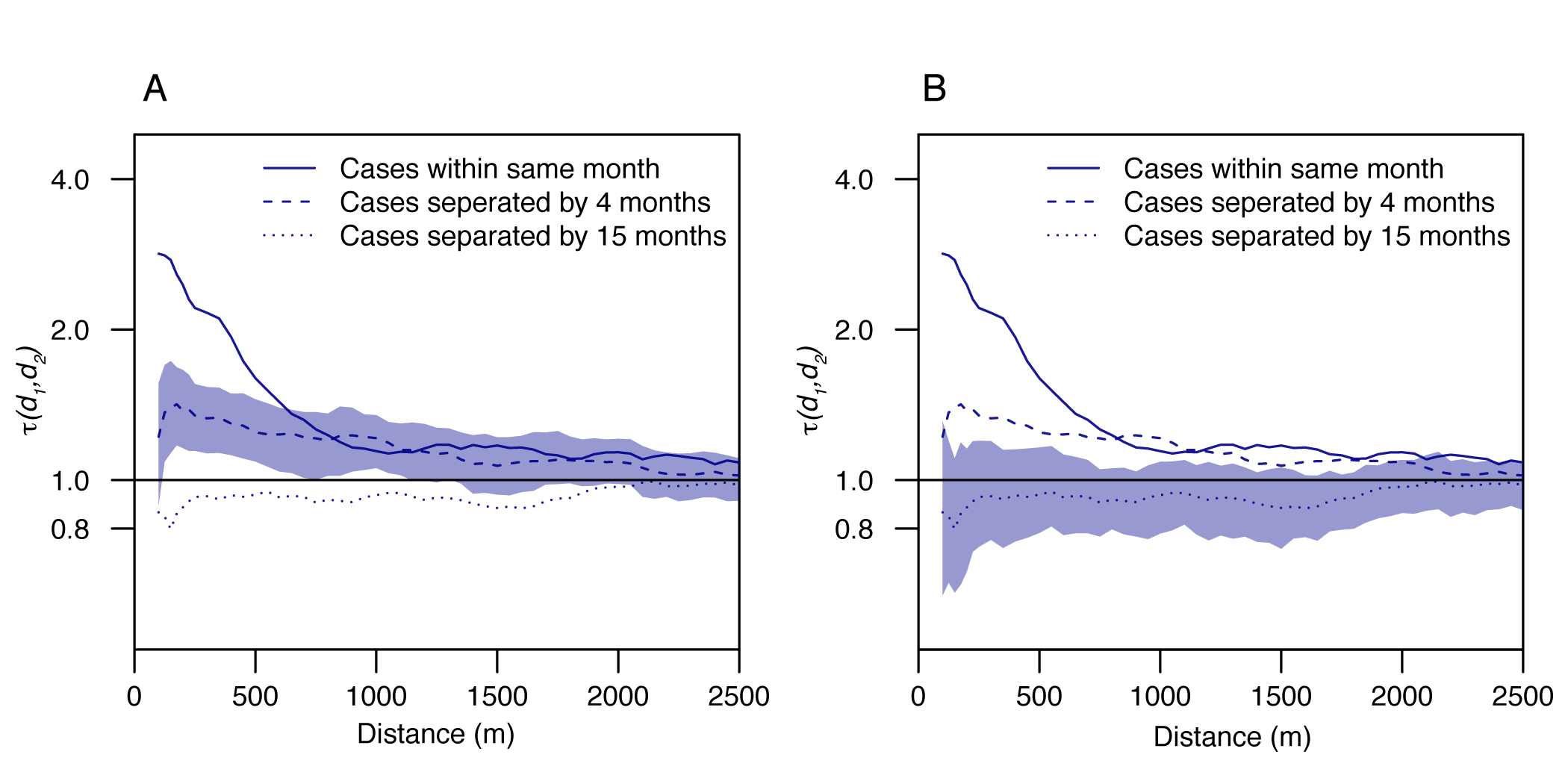

Supplement: S1 Fig — Confidence intervals for τ(d1, d2) estimates for cases separated by (A) four months and (B) 15 months. Results from 1,912 dengue cases that presented at a Bangkok hospital between 1995 and 1999. (TIF) [file pone.0155249.s001.tif]

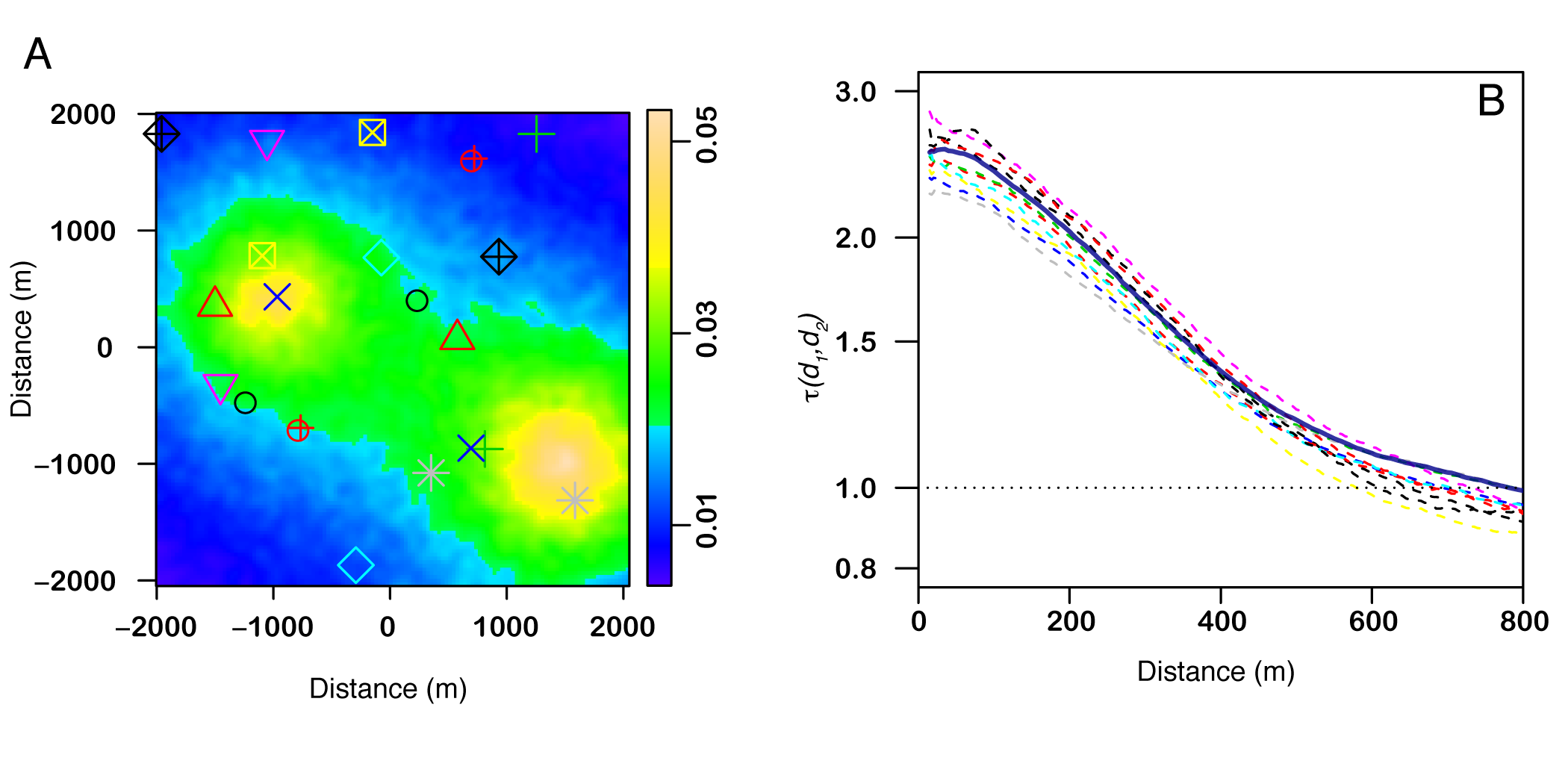

Supplement: S2 Fig — Estimates of τ(d1, d2) when there is spatially biased observation dependent on the location of a pair of healthcare locations. (A) Locations of pairs of healthcare providers. (B) Estimates of τ(d1, d2) using locations in (A). The color of each line is the color of the corresponding locations in (A). The solid blue line is the estimate when all cases are observed. (TIF) [file pone.0155249.s002.tif]
