# Supplementary material for: Measuring Spatial Dependence for Infectious Disease Epidemiology
Source: PLoS One. 2016 May 19;11(5):e0155249. doi: 10.1371/journal.pone.0155249 (PMC4873007; doi:10.1371/journal.pone.0155249)
Supplement: S1 Text — (PDF) [file pone.0155249.s003.pdf]

Supplementary appendices to "Measuring Spatial Dependence for Infectious Disease Epidemiology"

Justin Lessler, Department of Epidemiology, Johns Hopkins Bloomberg School of Public Health, Baltimore, MD
Henrik Salje, Department of Epidemiology, Johns Hopkins Bloomberg School of Public Health, Baltimore, MD
M. Kate Grabowski, Department of Epidemiology, Johns Hopkins Bloomberg School of Public Health, Baltimore, MD
Derek A. T. Cummings, Department of Epidemiology, Johns Hopkins Bloomberg School of Public Health, Baltimore, MD

Appendix 1 Proof that ratio of $\pi(d_1, d_2)$ and $\pi(\infty)$ represents the relative hazard of infection where underlying population is known

$\lambda_i(d_1, d_2)$: Infection hazard of a susceptible individual in distance range d_1, d_2 being infected by i
λ_i: Infection hazard of any susceptible individual being infected by i over all space (equivalent to $\lambda_i(\infty)$)
$S_i(d_1, d_2)$: Susceptible individuals in distance range d_1, d_2 of i
$P_i(d_1, d_2)$: Total population in distance range d_1, d_2 of i
$s_i(d_1, d_2)$: Proportion of population susceptible to infection in distance range d_1, d_2 of i

The hazard of a susceptible individual in distance range d_1, d_2 being infected by i can be calculated by $\pi(d_1, d_2)$:

$$\pi_{d_1,d_2} = \frac{\lambda_i(d_1, d_2) S_i(d_1, d_2)}{S_i(d_1, d_2)} = \lambda_i(d_1, d_2)$$

Therefore $\tau(d_1, d_2)$ represents the relative hazard:

$$\tau(d_1, d_2) = \frac{\pi(d_1, d_2)}{\pi(\infty)} = \frac{\lambda_i(d_1, d_2)}{\lambda_i}$$

When susceptibility is independent of the location of i:

$$\pi(d_1, d_2) = \frac{\lambda_i(d_1, d_2)s_i(d_1, d_2)P_i(d_1, d_2)}{P_i(d_1, d_2)} = \lambda_i(d_1, d_2)s_i(d_1, d_2)$$

$$\tau(d_1, d_2) = \frac{\pi(d_1, d_2)}{\pi(\infty)} = \frac{\lambda_i(d_1, d_2)s_i(d_1, d_2)}{\lambda_i s} = \frac{\lambda_i(d_1, d_2)}{\lambda_i} \text{ if } s = s_i(d_1, d_2)$$

Appendix 2: Edge corrections are not necessary when calculating $\tau(d_1, d_2)$

In addition to the definitions found in Appendix 1:
x_i: Probability of individual being in study area in distance range d_1, d_2 of i

If we assume there are no directional differences in the probability of an individual being a case, taking account of the probability of an individual being within the study area adjusts $\pi(d_1, d_2)$ as follows:

$$\pi(d_1, d_2) = \frac{\lambda_i(d_1, d_2)s_i(d_1, d_2)P_i(d_1, d_2)x_i}{P_i(d_1, d_2)x_i}$$

$$= \lambda_i(d_1, d_2)s_i(d_1, d_2)$$

Therefore the value for $\tau(d_1, d_2)$ is the same irrespective of the proportion of cases that are within the study area.

Appendix 3: Ratio of $\pi(d_1, d_2)$ and $\pi(\infty)$ biases $\tau(d_1, d_2)$ towards the null when underlying population is not known

When the underlying population distribution is not known and we instead use the distribution of typed case data to calculate $\pi(d_1, d_2)$, we get:

$$\pi_{d_1,d_2} = \frac{\lambda_i(d_1,d_2)s_i(d_1,d_2)P_i(d_1,d_2)}{\lambda_i(d_1,d_2)s_i(d_1,d_2)P_i(d_1,d_2) + \sum_{j\neq i}\lambda_j s_i(d_1,d_2)P_i(d_1,d_2)} = \frac{\lambda_i(d_1,d_2)}{\lambda_i(d_1,d_2) + \sum_{j\neq i}\lambda_j}$$

where i and j come from independent transmission chains (so λ_j does not depend on the location of i). The estimate of $\tau(d_1, d_2)$ therefore becomes:

$$\tau(d_1,d_2) = \frac{\pi(d_1,d_2)}{\pi(\infty)} = \frac{\lambda_i(d_1,d_2)}{\lambda_i(d_1,d_2) + \sum_{j\neq i}\lambda_j}\frac{\lambda_i + \sum_{j\neq i}\lambda_j}{\lambda_i}$$

$$= \frac{\lambda_i(d_1,d_2)}{\lambda_i}\frac{\lambda_i + \sum_{j\neq i}\lambda_j}{\lambda_i(d_1,d_2) + \sum_{j\neq i}\lambda_j}$$

as $\lambda_i(d_1, d_2) \geq \lambda_i \implies 1 \leq \tau(d_1, d_2) \leq \lambda_i(d_1, d_2)/\lambda_i$ and therefore $\tau(d_1, d_2)$ is biased towards the null.

Note that $\tau(d_1, d_2)$ is approximately equal to $\lambda_i(d_1, d_2)/\lambda_i$ if λ_i is close to zero or $\lambda_i(d_1, d_2)/\lambda_i$ is close to 1.

Appendix 4: Odds ratio gives unbiased estimate when underlying population not known

Define $\theta(d_1, d_2)$ as the odds ratio of cases related to those of individual i and those independent of i:

$$\theta_r = \frac{\lambda_i(d_1, d_2)s_i(d_1, d_2)P_i(d_1, d_2)}{\sum_{j \neq i} \lambda_j s_i(d_1, d_2)Pi(d_1, d_2)}$$

$$= \frac{\lambda_i(d_1, d_2)}{\sum_{j \neq i} \lambda_j}$$

In this scenario $\tau(d_1, d_2)$ becomes:

$$\tau(d_1, d_2) = \frac{\theta(d_1, d_2)}{\theta(\infty)} = \frac{\lambda_i(d_1, d_2)}{\sum_{j \neq i} \lambda_j} \frac{\sum_{j \neq i} \lambda_j}{\lambda_i}$$

$$= \frac{\lambda_i(d_1, d_2)}{\lambda_i}$$

which represents the relative hazard of infection.

Appendix 5. Estimator for $\tau(d_1, d_2)$ when underlying population distribution known

Where the underlying population distribution is known, we can estimate $\pi(d_1, d_2)$ as:

$$\hat{\pi}(d_1, d_2) = \frac{\sum_i \sum_j \mathbf{I}(z_{ij} = 1, d_1 < d_{ij} < d_2)}{\sum_k |\Omega_k(d_1, d_2)|}$$

where $\mathbf{I}$ is an indicator variable and is equal to one if cases i and j are potentially transmission related (as indicated by $z_{ij} = 1$) and are located within d_1 and d_2 of each other and is equal to zero otherwise; $|\Omega_k(d_1, d_2)|$ is the size of the underlying population within d_1 and d_2 of individual k.

The estimator for $\tau(d_1, d_2)$ is then:

$$\hat{\tau}(d_1, d_2) = \frac{\hat{\pi}(d_1, d_2)}{\hat{\pi}(0, \infty)}$$

Appendix 6. Estimator for $\tau(d_1, d_2)$ when underlying population distribution unknown

Where the underlying population distribution is not known, we instead estimate $\theta(d_1, d_2)$ using:

$$\hat{\theta}(d_1, d_2) = \frac{\sum_i \sum_j \mathbf{I_1}(z_{ij} = 1, d_1 < d_{ij} < d_2)}{\sum_i \sum_j \mathbf{I_2}(z_{ij} = 0, d_1 < d_{ij} < d_2)}$$

where $\mathbf{I_1}$ is an indicator variable and is equal to one if cases i and j are potentially transmission related (as indicated by $z_{ij} = 1$) and are located within d_1 and d_2 of each other and is equal to zero otherwise; $\mathbf{I_2}$ is an indicator variable and is equal to one if cases i and j can not be transmission related (as indicated by $z_{ij} = 0$) and are located within d_1 and d_2 of each other and is equal to zero otherwise.

The estimator for $\tau(d_1, d_2)$ in these circumstances is then:

$$\hat{\tau}(d_1, d_2) = \frac{\hat{\theta}(d_1, d_2)}{\hat{\theta}(0, \infty)}$$

Appendix 7. Proof that τ estimator is maximum likelihood estimate

The τ estimator describes the empirically observed probability ratios of particular types of case-pairs over varying distances. Let us consider y_k as a pair of cases, one of type i and one of type j that are d_k apart.

$$y_k =< i_k, j_k, d_k >$$

$$Y = \{y_1, y_2, ..., y_N\}$$

τ is written as:

$$\tau(d_1, d_2) = \frac{Pr(h(i,j)|d_1 < d_{ij} < d_2)}{Pr(h(i,j)|0 < d_{ij} < \infty)} = \frac{\pi(d_1, d_2)}{\pi(0, \infty)}$$

where $h(i, j)$ sets out whether i and j are transmission related. Assuming that pairs of cases are independent of each other. The likelihood of $\pi(d_1, d_2)$ can be written down as:

$$\mathcal{L}(\pi(d_1, d_2)) = \prod_k \left[(\pi(d_1, d_2))^{h(i_k,j_k)}(1 - \pi(d_1, d_2))^{1-h(i_k,j_k)}\right]^{\mathbf{I}(d_1<d_k<d_2)}$$

The log-likelihood is therefore:

$$l(\pi(d_1, d_2)) =$$

$$\sum_k \mathbf{I}(d_1 < d_k < d_2) \left[h(i_k, j_k) log(\pi(d_1, d_2)) + (1 - h(i_k, j_k)) log(1 - \pi(d_1, d_2))\right]$$

As all (i, j) pairs are included in k we can write it in terms of i and j:

$$= \sum_i \sum_j \mathbf{I}(d_1 < d_{ij} < d_2) \left[h(i, j) log(\pi(d_1, d_2)) + (1 - h(i, j)) log(1 - \pi(d_1, d_2))\right]$$

We can find the MLE by differentiating with respect to $\pi(d_1, d_2)$ and setting the result to 0:

$$dl/d\pi =$$

$$\sum_i \sum_j \mathbf{I}(d_1 < d_{ij} < d_2) \left[\frac{h(i,j)}{\pi(d_1, d_2)} - \frac{(1 - h(i,j))}{(1 - \pi(d_1, d_2))}\right] = 0$$

Therefore:

$$\frac{1-\pi(d_1,d_2)}{\pi(d_1,d_2)} = \frac{\sum_i \sum_j \mathbf{I}(d_1 < d_{ij} < d_2)(1-h(i,j))}{\sum_i \sum_j \mathbf{I}(d_1 < d_{ij} < d_2)h(i,j)}$$

$$\implies \pi(d_1,d_2) = \frac{\sum_i \sum_j \mathbf{I}(d_1 < d_{ij} < d_2)h(i,j)}{\sum_i \sum_j \mathbf{I}(d_1 < d_{ij} < d_2)}$$

which is our estimator for $\pi(d_1, d_2)$. The same derivation holds for $\pi(0, \infty)$. Therefore, through the property of invariance, as both the numerator and the denominator of $\tau(d_1, d_2)$ are MLEs, the ratio must also be the MLE.

Appendix 8: Demonstration that for bootstrapping need to avoid self comparisons when resampling

We can use a bootstrapping approach to calculate confidence intervals for the $\tau(d_1, d_2)$ estimates. We repeatedly resample the cases with replacement and calculate $\tau(d_1, d_2)$ after each resampling event. When calculating $\tau(d_1, d_2)$, we need to remove self-comparisons. Including them would bias $\tau(d_1, d_2)$ upwards by incorporating pairs of cases with 0 distance. The figure below sets out $\tau(d_1, d_2)$ estimate with confidence intervals where there is no removal of self-comparisons (blue) and where the adjustment is made (green). The bootstrap intervals were performed over 500 resampling events.

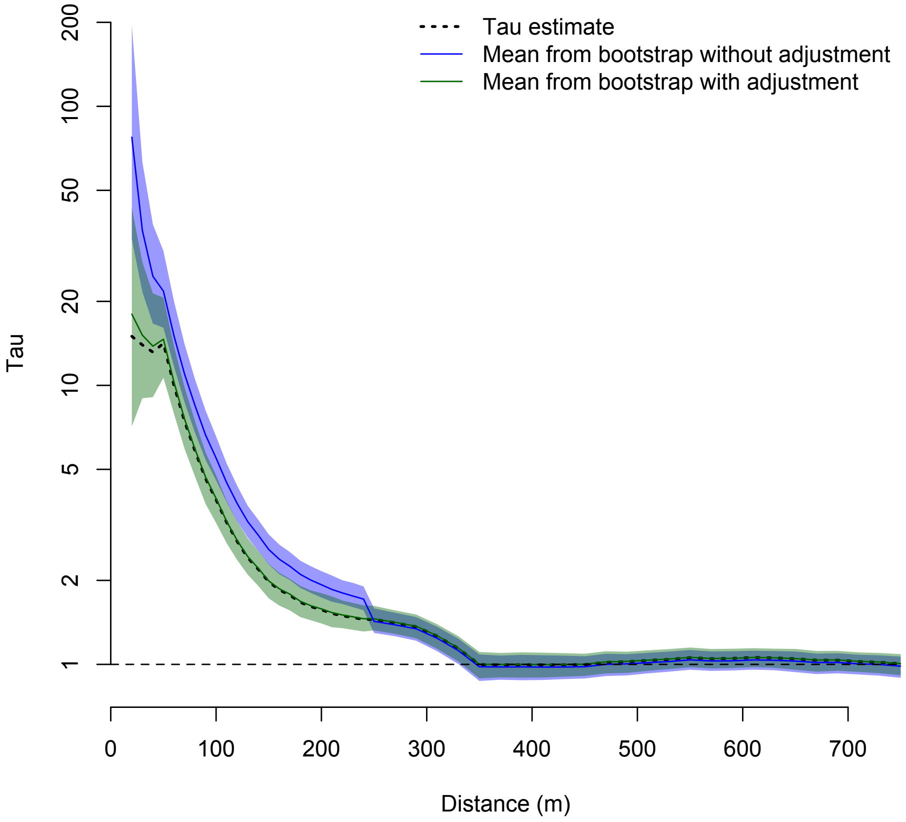

Appendix 9: Details on simulation studies

We explored the ability of our approach to estimate the relative hazard of infection at different distances and under different observation scenarios using simulation. We first generated a spatially inhomogeneous population of 320,000 individuals over a 16km2 area (Figure 2A) using a Matern cluster process with ten sub-populations, each of 32,000 individuals and mean radius of 1.4km. We then simulated a disease transmission process as follows: 100 randomly chosen individuals were infected, each with a separate strain. Each case then attempted to infect either one (in scenarios where R0=1) or two (in scenarios where R0=2) randomly chosen individuals. We built scenarios where infections were attempted in randomly selected individuals from the entire population (i.e., no spatial dependence in the transmission process) and where infections were only attempted in randomly selected individuals located within 100m of the infector. Infections were successful in individuals that hadnt previously been infected. All individuals were susceptible at the start of the simulation. The infected individuals then went on to infect further individuals. The process was repeated for ten generations. The time between successive infection events was held fixed at 15 days. The location, time and strain of each infection event were recorded. The 100 strains were also randomly divided into four equally sized serotype groups.

To explored the impact of incomplete observation under two different scenarios: (a) completely spatially random thinning and (b) spatially biased thinning. For (a) we randomly deleted 99% of all cases irrespective of where they occurred. For (b) we calculated the distance of each case from two 'surveillance hospitals' located at (-1000,0) and (1000,0) (see Figure 2A). Cases further away from the 'surveillance hospitals' would be less likely to be detected than ones further away (when they may turn up at different hospitals). The probability of detection for each case was calculated as 0.1*exp(-d), where d was the distance between the case and the closest surveillance hospital. In both observation scenarios, we calculated the ?-statistic using only the observed cases.
